# Directional Ion Transport Through Nanoarchitected 1D Mesochannels: 2D Polymer Interfacial Engineering for High‐Efficiency Capacitive Deionization

**DOI:** 10.1002/advs.202504527

**Published:** 2025-06-26

**Authors:** Chen Tang, Hongli Chen, Qian Li, Changle Li, Ying Li, Azhar Alowasheeir, Zeinhom M El‐Bahy, Guoxiu Wang, Chongyin Zhang, Yusuke Yamauchi, Xingtao Xu

**Affiliations:** ^1^ School of Chemistry and Chemical Engineering Shanghai Jiao Tong University 800 Dongchuan Road Shanghai 200240 China; ^2^ Centre for Clean Energy Technology School of Mathematical and Physical Science Faculty of Science University of Technology Sydney Sydney NSW 2007 Australia; ^3^ Marine Science and Technology College Zhejiang Ocean University Zhoushan Zhejiang 316022 China; ^4^ Shanghai Aerospace Equipments Manufacturer Co., Ltd 100 Huaning Road Shanghai 200245 China; ^5^ Department of Materials Process Engineering, Graduate School of Engineering Nagoya University Nagoya Aichi 464‐8603 Japan; ^6^ Department of Chemistry Faculty of Science Al‐Azhar University Nasr City Cairo 11884 Egypt; ^7^ Australian Institute for Bioengineering and Nanotechnology (AIBN) The University of Queensland Brisbane QLD 4072 Australia; ^8^ Department of Convergent Biotechnology & Advanced Materials Science Kyung Hee University 1732 Deogyeong‐daero, Giheung‐gu Yongin‐si Gyeonggi‐do 17104 South Korea

**Keywords:** 2D heterostructure, capacitive deionization, ion diffusion, mesochannel, polymer

## Abstract

The development of high‐performance capacitive deionization (CDI) electrodes demands innovative materials that integrate rapid ion transport, high salt adsorption capacity (SAC), and oxidative stability. This challenge is addressed through a surface nanoarchitectonics strategy, constructing 2D mesochannel polypyrrole/reduced graphene oxide heterostructures (mPPy/rGO) with ordered 1D mesochannels (~8 nm) parallel to the graphene surface. By confining the self‐assembly of cylindrical polymer brushes on freestanding rGO substrates, directional ion highways are simultaneously engineered that significantly reduce transport tortuosity. In addition, corrosion‐resistant polymer interfaces block oxygen penetration, and strong interfacial interactions between PPy and rGO ensure efficient electron transfer. The mPPy/rGO‐based CDI cell achieves breakthrough performance: ultrahigh SAC of 84.1 mg g^−1^ (4.5× activated carbon, the salt concentration: 2 g L^−1^), and 96.8% capacity retention over 100 cycles in air‐equilibrated saline solution (the salt concentration: 500 mg L^−1^). This interfacial confinement methodology establishes a universal paradigm for designing polymer‐based desalination materials with atomically precise transport pathways.

## Graphical abstract

The engineering 2D polymer/graphene heterostructures with aligned 1D mesochannels synergizes directional ion transport pathways, corrosion‐resistant interfaces, and rapid electron highways, overcoming traditional bottlenecks. The design scalability and tunability enable universal adaptation to diverse redox‐active polymers, while its mechanistic insights guide future optimization of high‐flow desalination systems. This work redefines electrode engineering paradigms, accelerating sustainable solutions for water desalination.

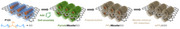

## 1 Introduction

The escalating global freshwater crisis demands electrochemical desalination technologies that harmonize rapid ion‐transport kinetics, large salt adsorption capacity (SAC), and oxidative stability—a trilemma unresolved by existing materials.^[^ ^1^ ^]^ Capacitive deionization (CDI), while energy‐efficient, suffers from intrinsic trade‐offs:^[^ ^2^ ^]^ porous carbon electrodes exhibit sluggish ion diffusion and limited SAC (<20 mg g^−1^), while redox‐active inorganic materials (e.g., MXenes, Prussian blue analogues, metal oxides) face metal dissolution and poor anion selectivity in saline electrolytes.^[^ ^3^ ^]^ Polymer‐based electrodes, with tunable redox sites and inherent corrosion resistance, offer a promising alternative.^[^ ^4^ ^]^ However, conventional synthesis methods yield structurally flawed systems:i) *π*–*π* stacking reduces accessible surface area, ii) tortuous micropores hinder ion transport, and iii) most polymers have high charge‐transfer resistance. In addition, hybridization with 2D conductors (e.g., graphene, MXenes) is attractive^[^ ^5^ ^]^ but often exacerbates anisotropic ion transport, failing to synchronize electron/ion dynamics.

Here, we address these challenges through surface mesochannel nanoarchitectonics, engineering 2D polymer/graphene heterostructures with aligned 1D nanochannels. As a proof of concept, mesochannel polypyrrole/reduced graphene oxide (mPPy/rGO) was synthesized, involving the self‐assembly of a cylindrical mesochannel PPy monolayer on graphene oxide (GO) nanosheets. The engineered mPPy/rGO exhibits several advantages, including directional ion highways that significantly reduce transport tortuosity, corrosion‐resistant polymer interfaces blocking oxygen penetration, and graphene‐polymer heterointerface ensuring efficient electron transfer, holding great potential for CDI application.

## 2 Results and Discussion

### 2.1 Synthesis and Characterization of mPPy/rGO

**Figure 1:**
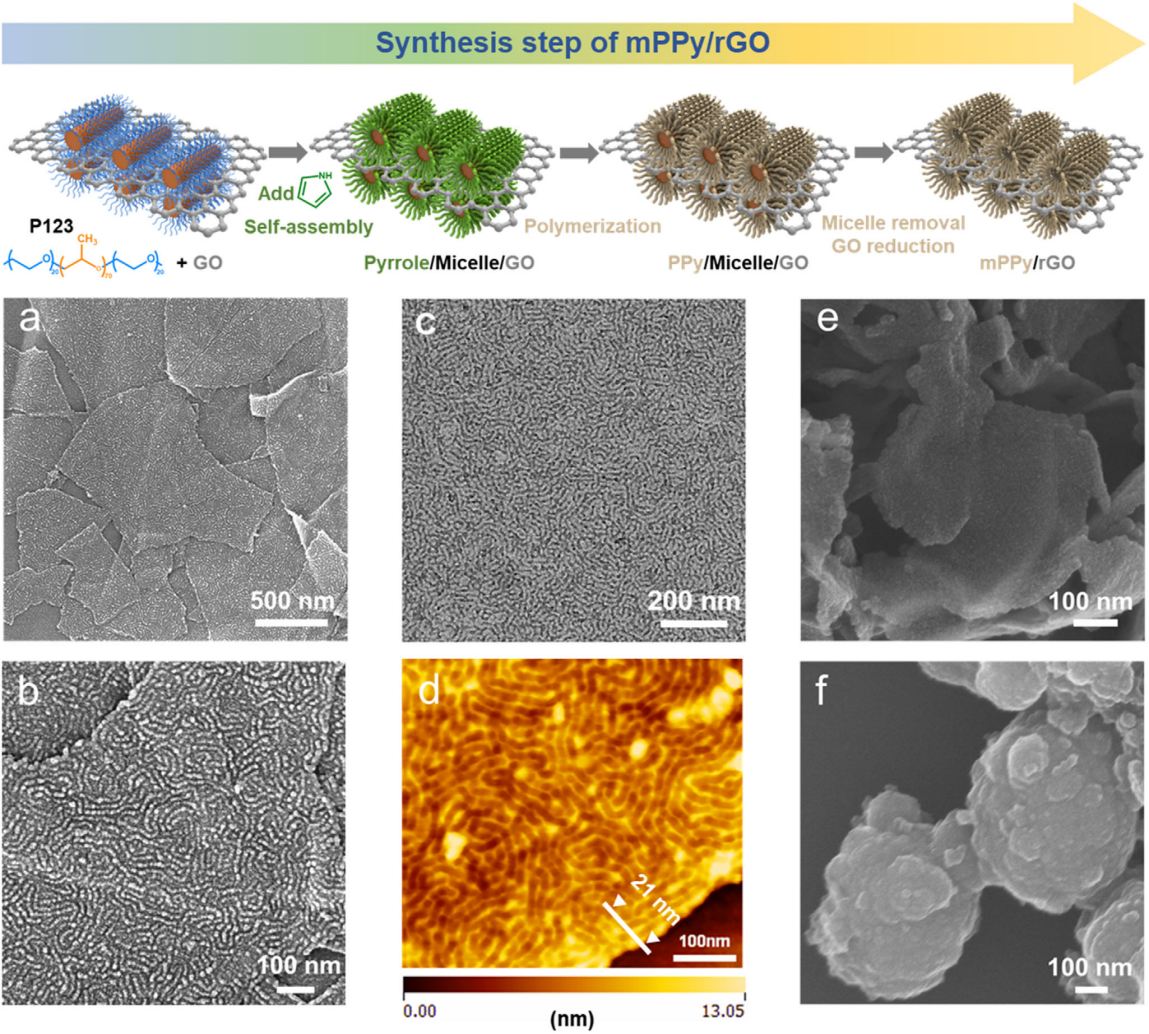
Synthetic procedure and morphological characterization of mPPy/rGO. a,b) SEM, c) TEM and d) AFM images of mPPy/rGO. SEM images of e) PPy/rGO and f) PPy nanoparticles. The white line with the triangle in (d) represents material thickness.

mPPy/rGO was synthesized by the self‐assembly of a cylindrical mesoporous PPy monolayer on GO nanosheets (**Figure** **1**). The GO nanosheets, prepared via Hummer's method,^[^ ^6^ ^]^ exhibit ultrathin, curly morphologies (Figure S1, Supporting Information). With Pluronic P‐123 (P123) copolymers as structure‐directing agents, the PPy monolayer with cylindrical mesopores is precisely patterned on 2D GO nanosheets, yielding a sheet‐like mPPy/rGO heterostructure devoid of individual nanoparticles (Figure 1a). High‐resolution scanning electron microscopy (SEM) and transmission electron microscopy (TEM) images reveal a 2D architecture with closely packed 1D mesochannels (Figure 1b,c), exhibiting a mean channel width of ~8 nm, consistent with the size of P123 cylindrical micelles.^[^ ^7^ ^]^

To further demonstrate the universality of this approach, polydopamine (PDA) mesochannels were similarly fabricated on rGO (mPDA/rGO), exhibiting identical structural features (Figure S2a–c, Supporting Information). Atomic force microscope (AFM) measurements confirm the presence of cylindrical mesochannels on the surface, with the heights of 21 nm for mPPy/rGO and 19 nm for mPDA/rGO (Figure 1d; Figure S2d, Supporting Information). 3D topographic AFM mappings of mPPy/rGO and mPDA/rGO are provided in Figure S3 (Supporting Information). To facilitate structural comprehension of the mPPy/rGO composite, Figure S4 (Supporting Information) provides schematic illustrations of the mPPy/rGO from both top and side views. Furthermore, conventional mechanical mixtures of rGO and PPy demonstrate pronounced phase segregation due to the lack of substantial interfacial interactions between the components (Figure S5, Supporting Information). Nitrogen adsorption–desorption analysis reveals a gradual uptake of nitrogen gas for mPPy/rGO (Figure S6, Supporting Information), characteristic of mesoporous materials, with a specific surface area of 403.1 m^2^ g^−1^, which is significantly higher than that of rGO (157.2 m^2^ g^−1^, Figure S7, Supporting Information). The Barrett–Joyner–Halenda (BJH) pore size distribution confirms an average mesopore diameter of ~8 nm, aligning with SEM/TEM observations. Because the mesoporous layer is very thin, the pore size peak is not clearly observed.

Through control experiments, the critical roles of GO and P123 were investigated. Without P123, PPy/rGO exhibits a smooth, nonporous surface (Figure 1e) and a reduced specific surface area (271.9 m^2^ g^−1^, Figure S8a, Supporting Information). In the absence of GO and P123, PPy forms rough, spherical nanoparticles with a negligible surface area (16.4 m^2^ g^−1^, Figure S8b, Supporting Information), highlighting the role of GO as a 2D template and P123 function as a pore‐forming agent. Fourier transform infrared spectroscopy (FTIR) spectra (Figure S9, Supporting Information) confirm successful PPy polymerization on rGO, with characteristic peaks at 1550 cm^−1^ (pyrrole ring stretching), 1470 cm^−1^ (C═C stretching), 1280 cm^−1^ (C─N stretching), and 1043 cm^−1^ (C─H bending).^[^ ^8^ ^]^

### 2.2 Electrochemical and Ion Adsorption Performances

The electrochemical properties of mPPy/rGO, PPy/rGO, and PPy were evaluated in a three‐electrode system with 1 m NaCl electrolyte. mPPy/rGO exhibits superior current density in cyclic voltammetry (CV) at 5 mV s^−1^ (Figure S10, Supporting Information), with well‐retained CV curves at scan rates up to 200 mV s^−1^ (Figure S11, Supporting Information), indicating high electrochemical reversibility. Specific capacitances (*C*_s_) calculated from CV reveal mPPy/rGO's dominance across all scan rates (Figure S12, Supporting Information), and galvanostatic charge–discharge (GCD) curve achieves 269 F g^−1^ at 0.5 A g^−1^—surpassing PPy/rGO (251 F g^−1^) and PPy (210 F g^−1^) (Figure S13, Supporting Information). Electrochemical impedance spectroscopy (EIS) further validates the advantages of mPPy/rGO, showing a lower charge‐transfer resistance (*R*_ct_ = 19.3 Ω) compared to PPy/rGO (41.6 Ω) and PPy (56.8 Ω) (Figure S14, Supporting Information). The steeper slope in the low‐frequency region of the Nyquist plot (Figure S15, Supporting Information) confirms faster Cl^−^ diffusion in mPPy/rGO, attributable to its mesochannel structure and enhanced electrolyte‐electrode contact.^[^ ^9^ ^]^

**Figure 2:**
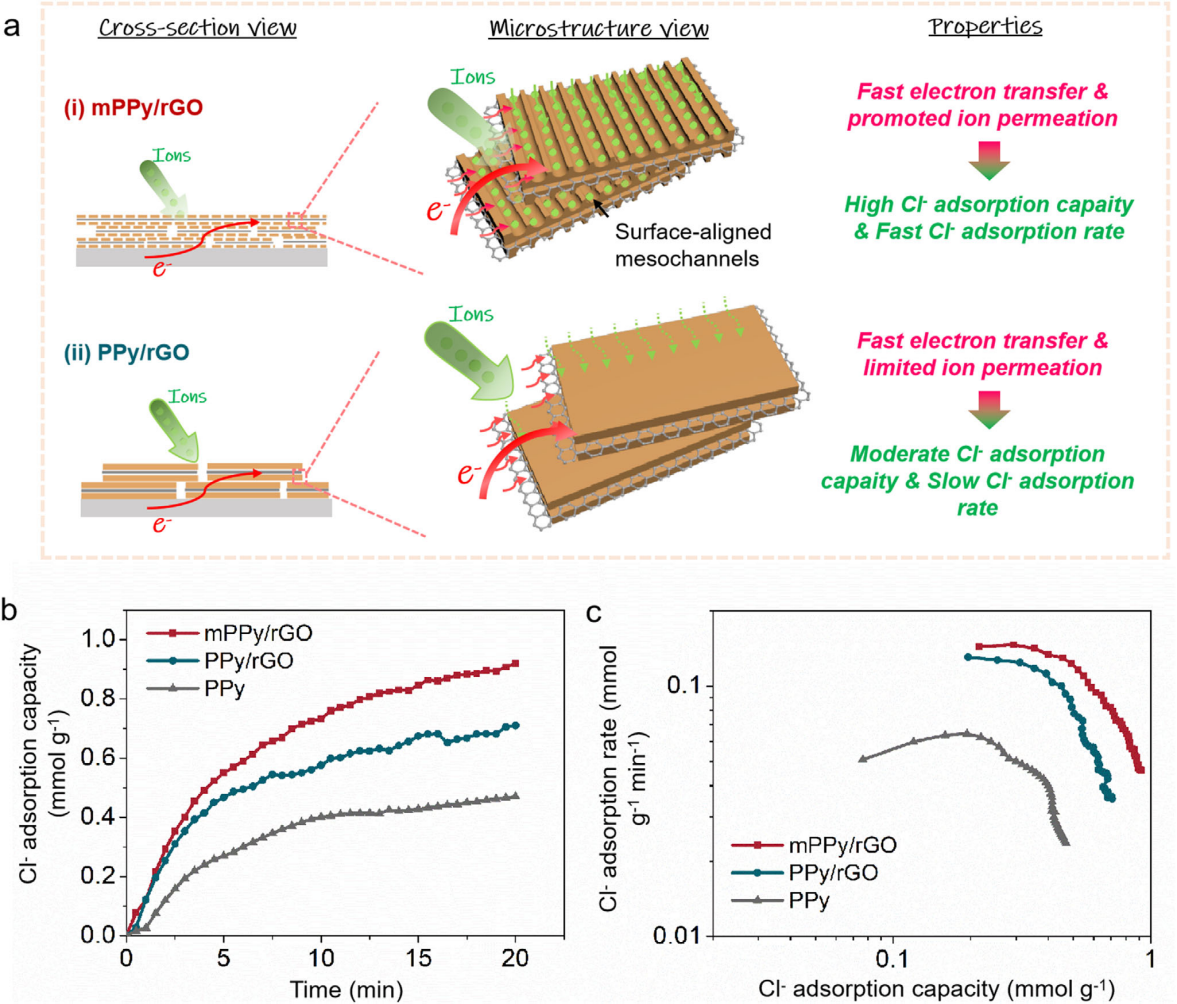
a) Scheme of ion and electron transfer pathways within i) mPPy/rGO and ii) PPy/rGO. b) Cl^−^ adsorption capacity variations and c) CDI Ragone plots of mPPy/rGO, PPy/rGO and PPy.

The unique architecture of mPPy/rGO features 1D mesochannels and 2D conductive support, which facilitates rapid ion and electron transport (**Figure** **2**a(i)), contrasting sharply with the limited pathways in PPy/rGO (Figure 2a(ii)) and PPy (Figure S16, Supporting Information). The schematic diagram illustrating the individual components of the CDI device is presented in Figure S17 (Supporting Information), with comprehensive descriptions provided in the corresponding figure caption. In CDI tests at 1.2 V, mPPy/rGO achieves a Cl^−^ adsorption capacity of 0.92 mmol g^−1^ in 100 mg L^−1^ NaCl, outperforming PPy/rGO (0.71 mmol g^−1^) and PPy (0.47 mmol g^−1^) (Figure 2b). The Cl^−^ adsorption rate of mPPy/rGO reaches 0.046 mmol g^−1^ min^−1^, significantly faster than PPy/rGO (0.036 mmol g^−1^ min^−1^) and PPy (0.024 mmol g^−1^ min^−1^) (Figure 2c).

### 2.3 Mechanism Insight into CDI Process

**Figure 3:**
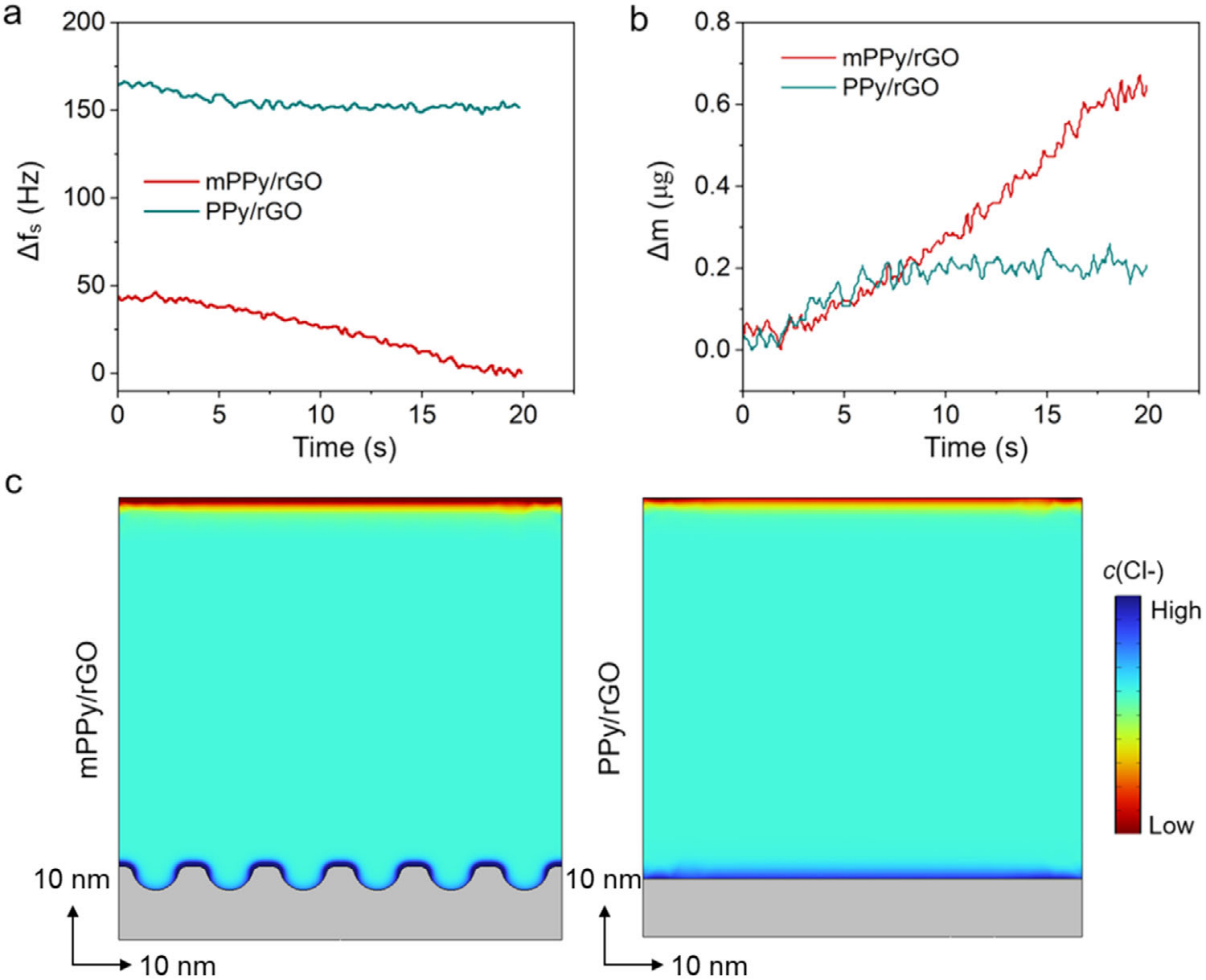
*In*‐*situ* EQCM analysis of mPPy/rGO and PPy/rGO in 1000 mg L^−1^ NaCl aqueous solution using CV in the voltage range of −0.5–0.5 V, with a) *f*s response and b) electrode mass changes calculated from the *f*s response. c) Finite element analysis of mPPy/rGO and PPy/rGO in terms of Cl^−^ concentration.

To quantify mass transport during Cl^−^ adsorption, in‐situ electrochemical quartz crystal microbalance (EQCM) measurements were carried out, which correlate changes in electrode mass with crystal oscillation frequency via the Sauerbrey equation.^[^ ^10^ ^]^ During CV at 50 mV s^−1^ in 1000 mg L^−1^ NaCl, the EQCM response of mPPy/rGO closely tracks the charging process (**Figure** **3**a). Notably, mPPy/rGO exhibits the larger frequency shift, corresponding to its higher Cl^−^ adsorption capacity. In addition, the adsorbed weight change of mPPy/rGO is also higher (Figure 3b). These results demonstrate that the mesochannel architecture of mPPy/rGO enhances both Cl^−^ adsorption kinetics and capacity by optimizing ion transport pathways and interfacial charge transfer. Furthermore, the Cl^−^ distributions within the single‐side surface of mPPy/rGO and PPy/rGO heterostructures after charging were investigated by finite element analysis (Figure 3c). It is proved that mPPy/rGO with surface‐aligned mesochannels, referring to more rough surface, has more contact with Cl^−^. In other words, more Cl^−^ are distributed around mPPy/rGO surface, indicating enhanced Cl^−^ adsorption kinetics and capacity due to surface‐aligned mesochannel architecture.

### 2.4 CDI Performance Evaluation

**Figure 4:**
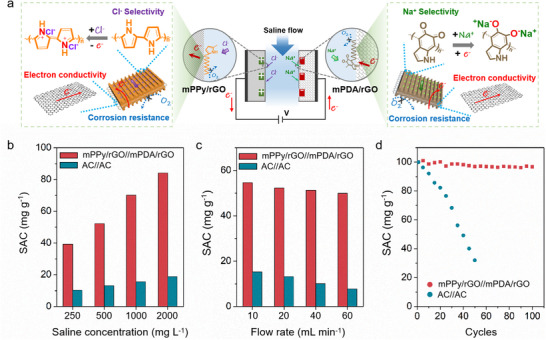
a) Scheme of a CDI process comprising mPPy/rGO//mPDA/rGO. b) SACs at different saline concentrations (Voltage: 1.2 V, Flow rate: 20 mL min^−1^), c) SACs at different flow rates (Voltage: 1.2 V, Concentration: 500 mg L^−1^), and d) SAC retention during cycles.

The practicality of mPPy/rGO was further evaluated by assembling mPPy/rGO as anode and mPDA/rGO as cathode into one CDI cell (**Figure** **4**a), which was further compared with commercial activated carbon (AC)‐based CDI benchmark. With the increase of saline concentrations (Voltage: 1.2 V, Flow rate: 20 mL min^−1^), the SAC of mPPy/rGO improves accordingly, surpasses AC at any concentrations, and reaches a record value of 84.1 mg g^−1^ at 2000 mg L^−1^ NaCl solution (Figure 4b). This performance surpasses many other CDI electrodes (Table S1, Supporting Information). The increase of operation voltage from 0.9 to 1.5 V (Concentration: 500 mg L^−1^ NaCl, Flow rate: 20 mL min^−1^) yields the increased SAC of mPPy/rGO, higher than those of AC (Figure S18, Supporting Information). When further increasing the flow rate (Concentration: 500 mg L^−1^ NaCl, Voltage: 1.2 V), mPPy/rGO also demonstrates excellent SAC retention (Figure 4c), showing the potential of mesochannel architecture on 2D interface for high‐flow desalination. The cycling stability measurement of mPPy/rGO exhibits 96.8% of its initial capacity after 100 cycles in air‐equilibrated saline water (Concentration: 500 mg L^−1^ NaCl, Voltage: 1.2 V, Flow rate: 20 mL min^−1^), significantly exceeding AC (Figure 4d).

## 3 Conclusion

In conclusion, we have demonstrated 2D interfacial nanoarchitectonics to construct mPPy/rGO with aligned 1D mesochannels (width: ~8 nm), resolving ion/electron transport trade‐offs in CDI. The engineered nanochannels efficiently reduce tortuosity for ultrafast Cl^−^ adsorption (0.046 mmol g^−1^ min^−1^), while oxygen‐blocking interfaces ensure 96.8% capacity retention over 100 cycles. Operando EQCM and finite element analysis reveal enhanced Cl^−^ adsorption kinetics and capacity. Achieving record SAC (84.1 mg g^−1^, 2000 mg L^−1^ NaCl) with universal scalability (mPDA/rGO), this work pioneers a materials‐by‐design paradigm for high‐flow desalination.

## Conflict of Interest

The authors declare no conflict of interest.

## Supporting information



Supporting Information

## Data Availability

The data that support the findings of this study are available from the corresponding author upon reasonable request.
