# Supplementary material for: Directional Ion Transport Through Nanoarchitected 1D Mesochannels: 2D Polymer Interfacial Engineering for High‐Efficiency Capacitive Deionization
Source: Adv Sci (Weinh). 2025 Jun 26;12(34):e04527. doi: 10.1002/advs.202504527 (PMC12442647; doi:10.1002/advs.202504527)
Supplement: Supplementary file 1 — Supporting Information [file ADVS-12-e04527-s001.docx]

Supporting Information

**Directional Ion Transport through Nanoarchitected 1D Mesochannels: 2D Polymer Interfacial Engineering for High-Efficiency Capacitive Deionization**

Chen Tang, Hongli Chen, Qian Li, Changle Li, Ying Li, Azhar Alowasheeir, Zeinhom M El-Bahy, Guoxiu Wang, Chongyin Zhang,* Yusuke Yamauchi,* Xingtao Xu*

**S1. Experimental Section**

**Chemicals and materials**

Graphite flakes, Pluronic^®^ P-123 (P123), pyrrole, ammonium persulfate (APS), ethanol, NaCl, Super P, polyvinylidene fluoride (PVDF), *N*-methyl–2–pyrrolidone (NMP), tris(hydroxymethyl)aminomethane (Tris) and tetrahydrofuran (THF) were purchased from Sigma–Aldrich.

**Synthesis of graphene oxide (GO)**

GO was synthesized from graphite flakes by using modified Hummers method. The concentration of GO aqueous dispersion is 0.5 mg mL^–1^.

**Synthesis of mPPy/rGO**

The mPPy/rGO was prepared based on self–assembly of block copolymer on GO, forming a template for polymerization of pyrrole. The P123 (100 mg) was first dissolved in deionized water (40 mL). After stirring for two hours at 40 ºC, GO solution (9 mL, 0.5 mg mL^–1^) and HCl (3 mL, 0.5 M) were added to the mixture. The mixture was stirred for two hours at 40 ºC, followed by adding pyrrole (50 µL) into the solution and continuously stirring for two hours. Then, aqueous solution of ammonium persulfate (10 mL, 19 mg mL^–1^) was dropped into the mixture to initiate the polymerization of pyrrole and react for 12 hours. Afterward, mPPy/GO was obtained after washing (with ethanol, THF and water) for several times and centrifugation. Finally, mPPy/GO was transferred to autoclaves for hydrothermal treatment at 180 ºC for 12 hours to obtain mPPy/rGO.

**Synthesis of PPy/rGO and PPy**

The synthesis of PPy/rGO followed a procedure similar to that applied to mPPy/rGO except for the addition of P123. PPy was synthesized without P123 and GO.

**Synthesis of mPDA/rGO**

The synthesis of mPDA/rGO was similar to that of mPPy/rGO. 100 mg of P123 was first dissolved in deionized water (40 mL). After stirring for two hours at 40 ºC, GO solution (9 mL, 0.5 mg mL^–1^), HCl (3 mL, 0.5 M) and 100 mg of Tris were added to the mixture. The mixture was stirred for two hours at 40 ºC, followed by adding 200 mg of dopamine hydrochloride into the solution and continuously stirring for two hours. The subsequent experimental procedures were the same as for mPPy/rGO.

**Characterization**

Transmission electron microscopy (TEM) studies were performed using a JEOL JEM–2100 microscope equipped with a LaB6 gun operated at 200 kV (Cs 1.0 mm, point resolution of 2.3 Å). Scanning electron microscopy (SEM) observations were performed on Hitachi SU8000 (5 kV) microscope. Nitrogen adsorption isotherms were measured using a BELSORP–mini (BEL, Japan) at 77 K. Before measurement, samples were degassed in a vacuum at 150 ºC for at least six hours. Brunauer–Emmett–Teller (BET) method was utilized to calculate the specific surface area using adsorption data in a relative pressure range from 0.05 to 0.5. The pore size distributions and pore volumes were derived from the adsorption branches of isotherms using Barrett–Joyner–Halenda (BJH) method. Fourier transform infrared (FTIR) spectroscopy was performed using the Thermoscientific Nicolet 4700 spectrometer.

**Electrochemical measurements**

The cyclic voltammetry (CV), galvanostatic charge-discharge (GCD) and electrochemical impedance spectrum (EIS) measurements were conducted on a CHI 842B electrochemical analyzer (CH Instrument, USA) using a three–electrode system with aqueous 1 M NaCl solution, platinum wire and standard calomel electrode as the electrolyte, counter electrode and reference electrode, respectively. The working electrode was prepared as follows: Firstly, 24 mg of the active material, 3 mg of the carbon black (Super P) and PVDF were dispersed in 1.5 mL of NMP solvent. After ultrasonication for one hour, 125 μL of the mixture is dropped onto a graphite paper (area of 1 × 2 cm^2^) and dried at 60 ºC. The specific capacitance (*C*, F g^-1^) can be obtained from the CV and GCD curves according to the following equations (1) and (2), respectively:

$C={\int IdV}/{2v\Delta Vm}$ (1)

$C=i\Delta t$ (2)

where *I* (A) is the current, *ΔV* (V) is the potential window, *v* (V s^–1^) is the scan rate, *m* is the total mass loading of the active materials (g), *i* (A g^-1^) is the current density, and *ΔV* (s) is the discharging time.

**CDI measurements**

The CDI performance of electrode materials was evaluated on a single CDI device. The CDI electrodes were prepared as followed: active material, super P and PVDF with the weight ratio of 8:1:1 was mixed in NMP solvent to form a slurry. Then, the slurry was dropped onto graphite paper (2.5 × 2.5 cm^2^) and dried at 60 ℃ for 12 h. Each CDI cell comprises a pair of identical electrodes. Batch–mode desalination experiments were conducted in aqueous NaCl solution with a continuous recycling system, including an CDI apparatus, a peristaltic pump, a power source, and a tank. In each experiment, the real-time saline concentration, current, and pH variation were monitored and measured at the outlet of the CDI apparatus. The volume of the saline solution was fixed at 30 mL, the flow rate was maintained at 20 mL min^–1^, and the operating voltage was 1.2 V. The Cl^-^ adsorption capacity (mmol g^–1^) and rate at *t* min (mmol g^–1^ min^–1^), and salt adsorption capacity (*SAC*, mg g^–1^) were calculated using the following equations:

${Cl}^{-} adsorption capacity={(C_{0}-C_{t})\times V}/{Mm_{cathode}}$ (3)

${Cl}^{-} adsorption rate={{Cl}^{-} adsorption capacity}/t$ (4)

$SAC={(C_{0}-C_{t})\times V}/m$ (5)

where *C*_0_ and *C*_t_ represent the concentrations of NaCl at initial stage and *t* min, respectively; *V* represents the volume of the NaCl solution (L); *M* is molar mass of NaCl (58.44 g mol^–1^); and *m*_cathode_ and *m* represent the mass of the active material onto the cathode and both-side electrodes, respectively (g).

**In-situ EQCM measurements**

In-situ electrochemical quartz crystal microbalance (EQCM, 10M, Gamry Instruments) measurements were performed using an AT-cut quartz crystal resonator equipped with a gold disk electrode (Active area: 0.79 cm^2^), operating at a fundamental frequency of 5.0 MHz. A saturated Ag/AgCl electrode served as the reference electrode, while a platinum foil acted as the counter electrode. The test electrolyte in the EQCM measurement system was a 500 mg/L NaCl solution.

**Finite element simulations**

In order to simulate the electric field and chlorine ion density within the vicinity of the electrodes, a finite element analysis (FEA) model was performed with the “Electrostatics” and “Transport of Diluted Species” modules. The electric field (*E*) was computed as the opposite gradient of the electric potential (*V*) as follows:

$E=-\nabla V$ (6)

The charge density (*ρ*) was computed via Gauss’s law for the electric field as follows:

$\rho=\varepsilon_{r}\varepsilon_{0}\nabla\cdot E$ (7)

where $\varepsilon_{0}$ denotes the dielectric function for a vacuum and $\varepsilon_{r}$ denotes the dielectric function of the materials.

In this work, the EDL was simulated via the Gouy-Chapman-Stern model, which comprises a Helmholtz layer and a diffusion layer. The Helmholtz layer encompasses a monolayer of surface-adsorbed hydrated anions on the electrode surface. The diffusion layer includes both cations and anions, which freely diffuse in the electrolyte and form concentration gradients towards and away from the electrode surface. The diffusion layer was established as the result of a dynamic equilibrium between electrostatic forces and diffusion (entropic forces). The “Electrostatics” and “Transport of Diluted Species” modules were combined to determine the chlorine ion density in the EDL. The Poisson-Nerst-Planck equations were solved in the steady state:

$\nabla^{2}V=\left\{ \begin{aligned} 0, &d<d_{H} \\ \left( c_{Cl}-c_{Na} \right)F, &d>d_{H} \end{aligned} \right.$ (8)

$\nabla\cdot\left( D\nabla c_{i}+\frac{Dz_{i}e}{k_{B}T}c_{i}\nabla V \right)=0$ (9)

where *d* is the distance from the electrode surface into the electrolyte and *d_H_* is the thickness of the Helmholtz layer, which is chosen as the radius of a hydrated chlorine ion. Notably, *d* < *d_H_* within the Helmholtz layer and *d* > *d_H_* within the diffusion layer. Moreover, *c_i_* (*i* ∈ {Na^+^, Cl^−^}) denotes the concentration of sodium or chlorine ions, *z_i_* is the valency of both ions, *e* is the elementary charge, *k_B_* is the Boltzmann constant, *D* is the diffusion coefficient, *F* is the Faraday constant, and the absolute temperature *T* was set to 297.3 K.

**S2. Supporting Figure S1–S18**


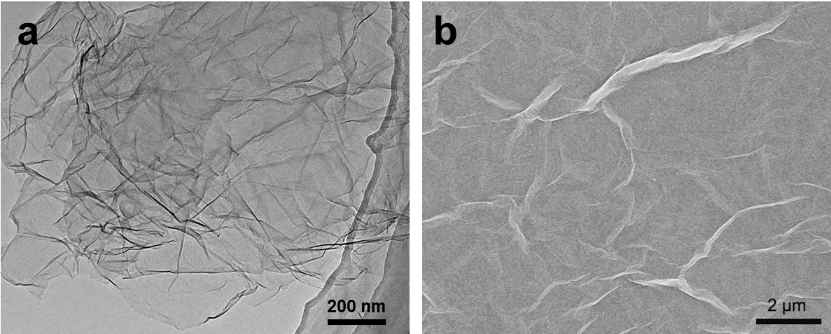


**Figure S1.** (a) TEM and (b) SEM images of GO nanosheets.


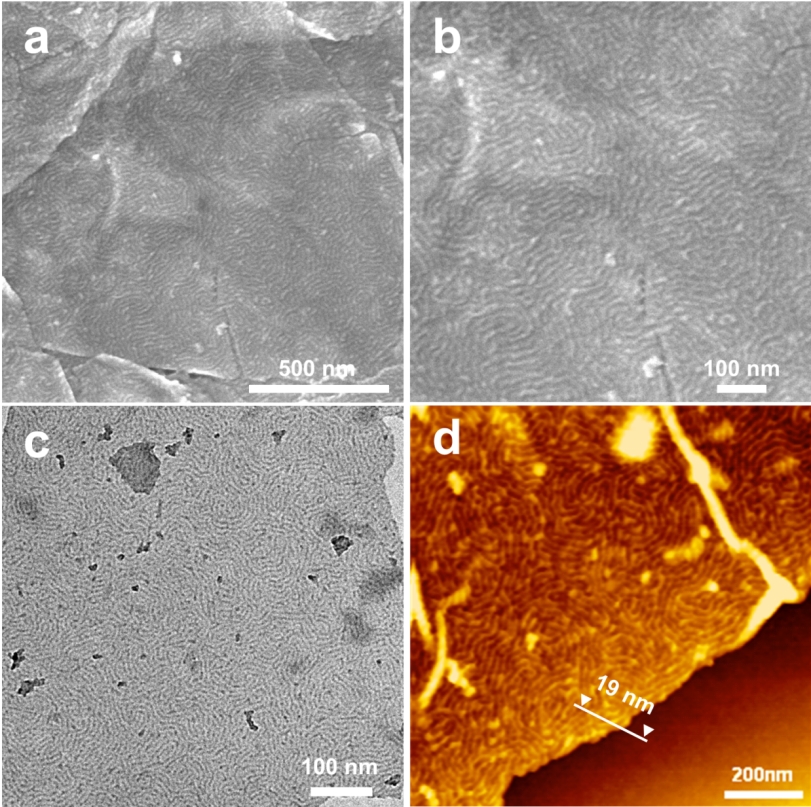


**Figure S2.** (a, b) SEM, (c) TEM, and (d) AFM images of mPDA/rGO (the white line with the triangle represents material thickness).

**
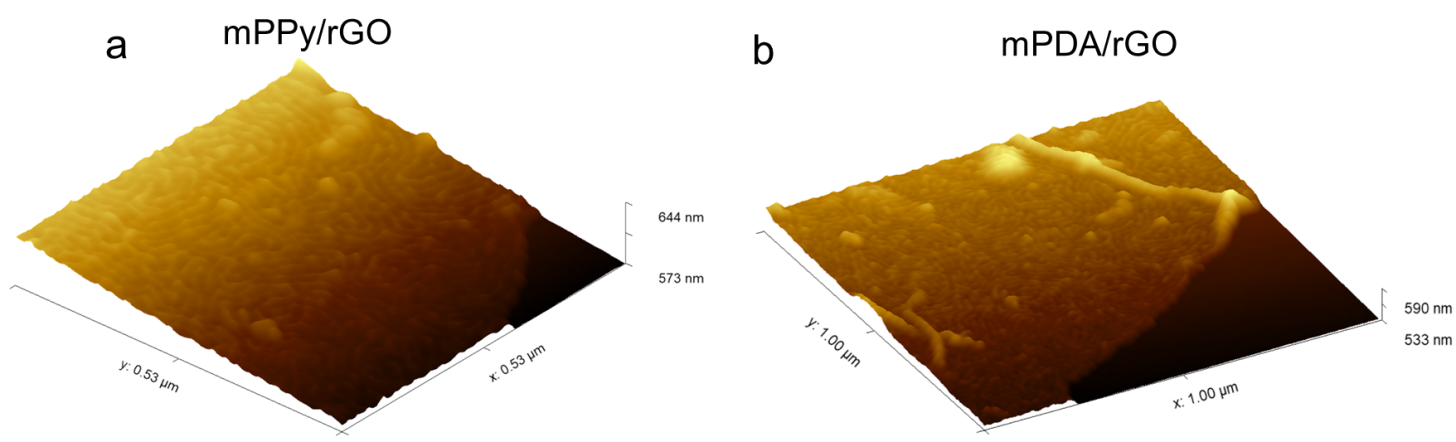
**

**Figure S3.** 3D topography of (a) mPPy/rGO and (b) mPDA/rGO.

**
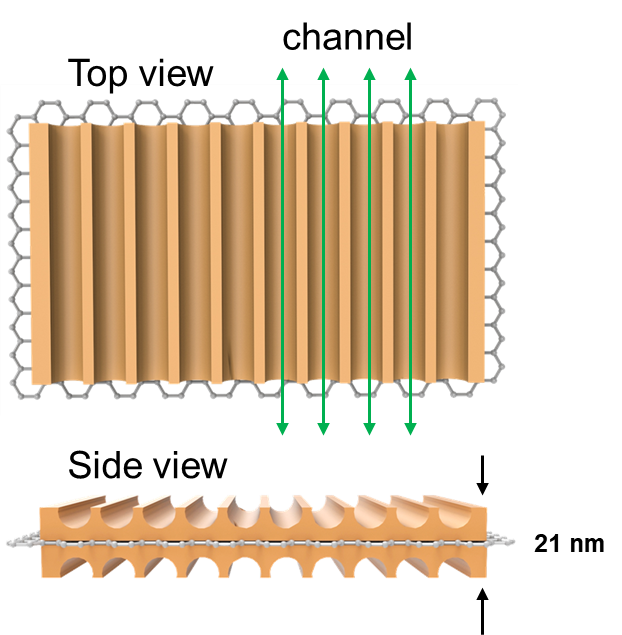
**

**Figure S4.** Schematic illustrations of the mPPy/rGO from top and side views.


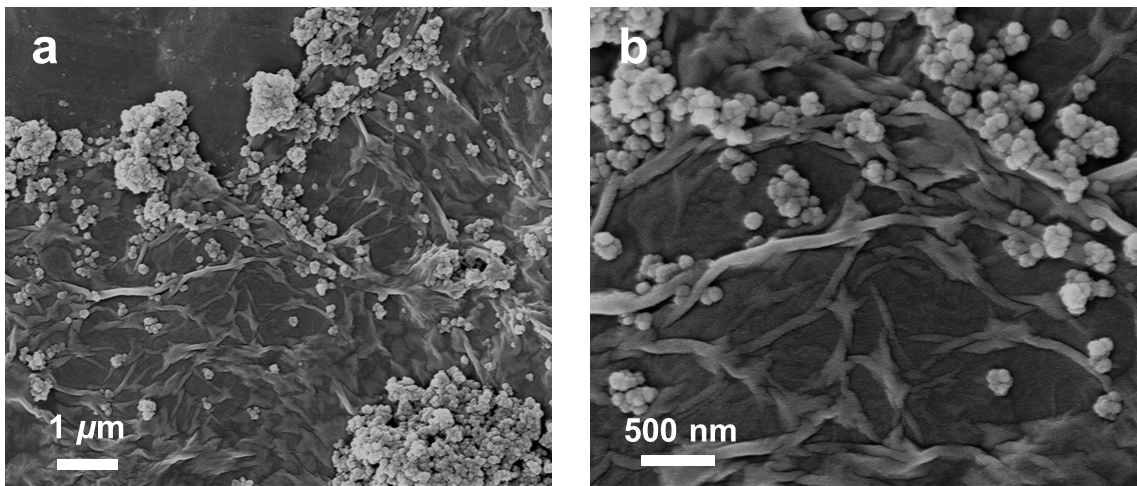


**Figure S5.** (a) Low-magnification and (b) high-magnification SEM images of the physical mixture of rGO and PPy.


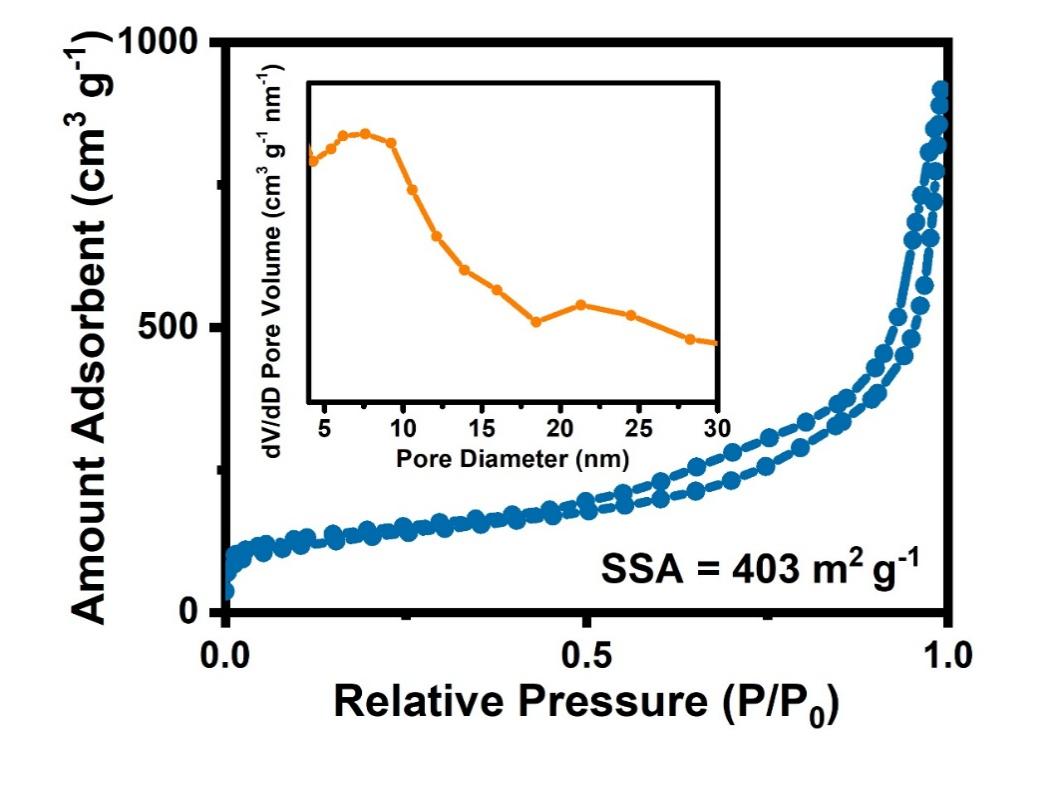


**Figure S6.** Nitrogen adsorption–desorption isotherms and pore size distributions of mPPy/rGO.


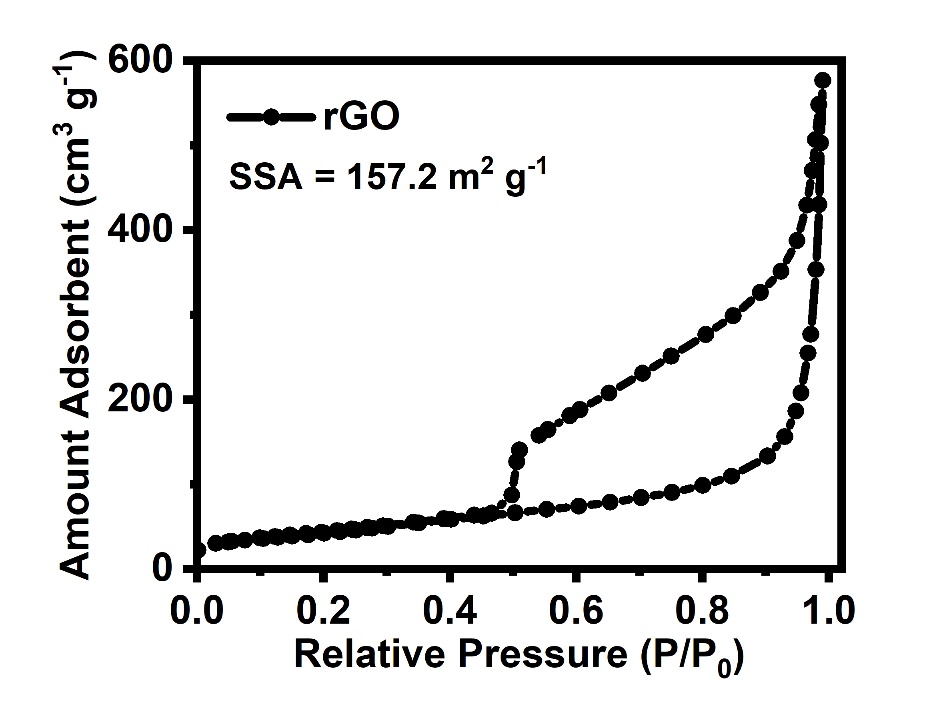


**Figure S7**. Nitrogen absorption–desorption isotherms of rGO.


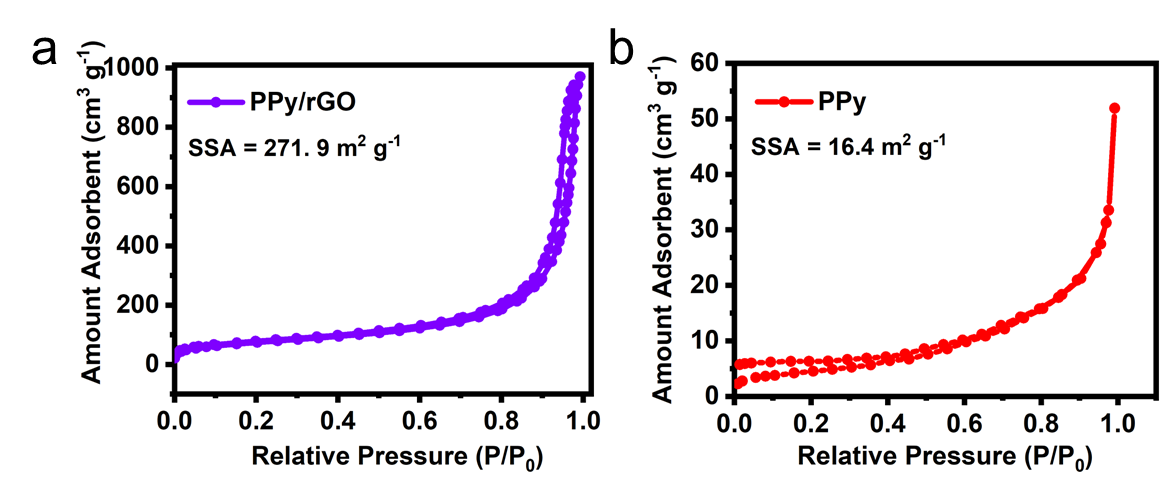


**Figure S8.** Nitrogen absorption–desorption isotherms of (a) PPy/rGO and (b) PPy.


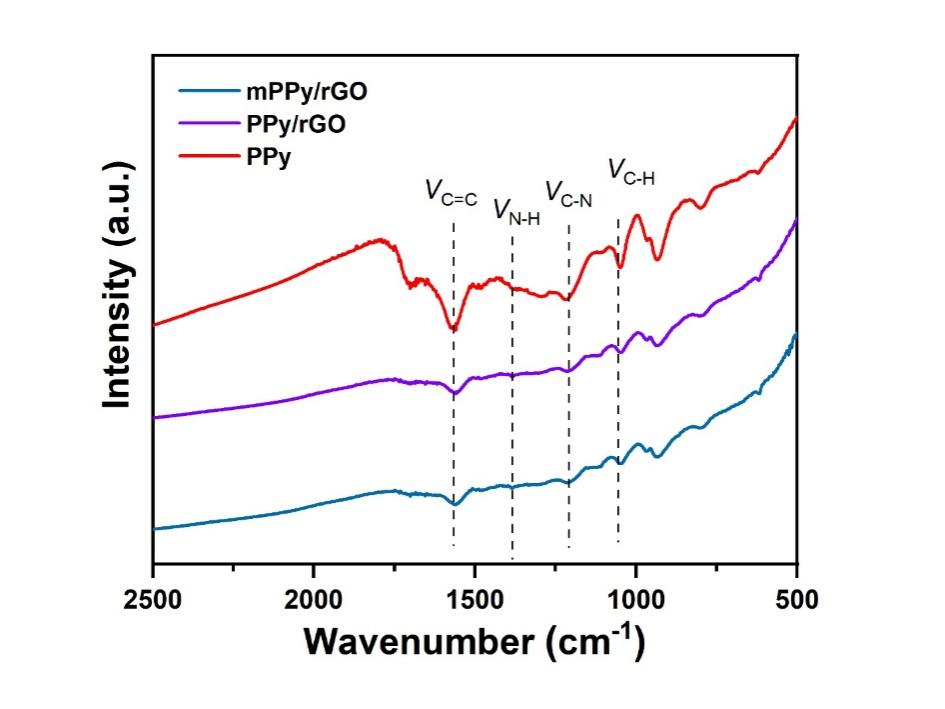


**Figure S9.** FTIR spectra of mPPy/rGO, PPy/rGO and PPy. The peak at 1572 cm^-1^ is assigned to C=C bond of pyrrole. And the peak at 1383 cm^-1^ is associated with N-H deformation of the pyrrole ring. The peaks at 1212 and 1057 cm^-1^ are ascribed to C-N and C-H groups, respectively.


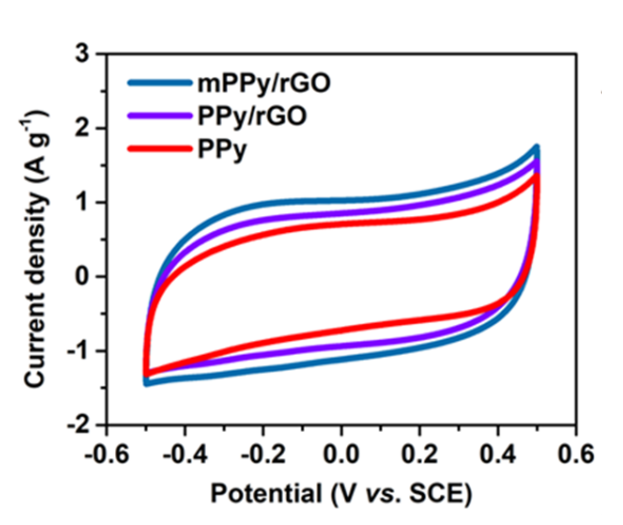


**Figure S10.** CV curves of mPPy/rGO, PPy/rGO and PPy collected at the scan rate of 5 mVs^–1^.


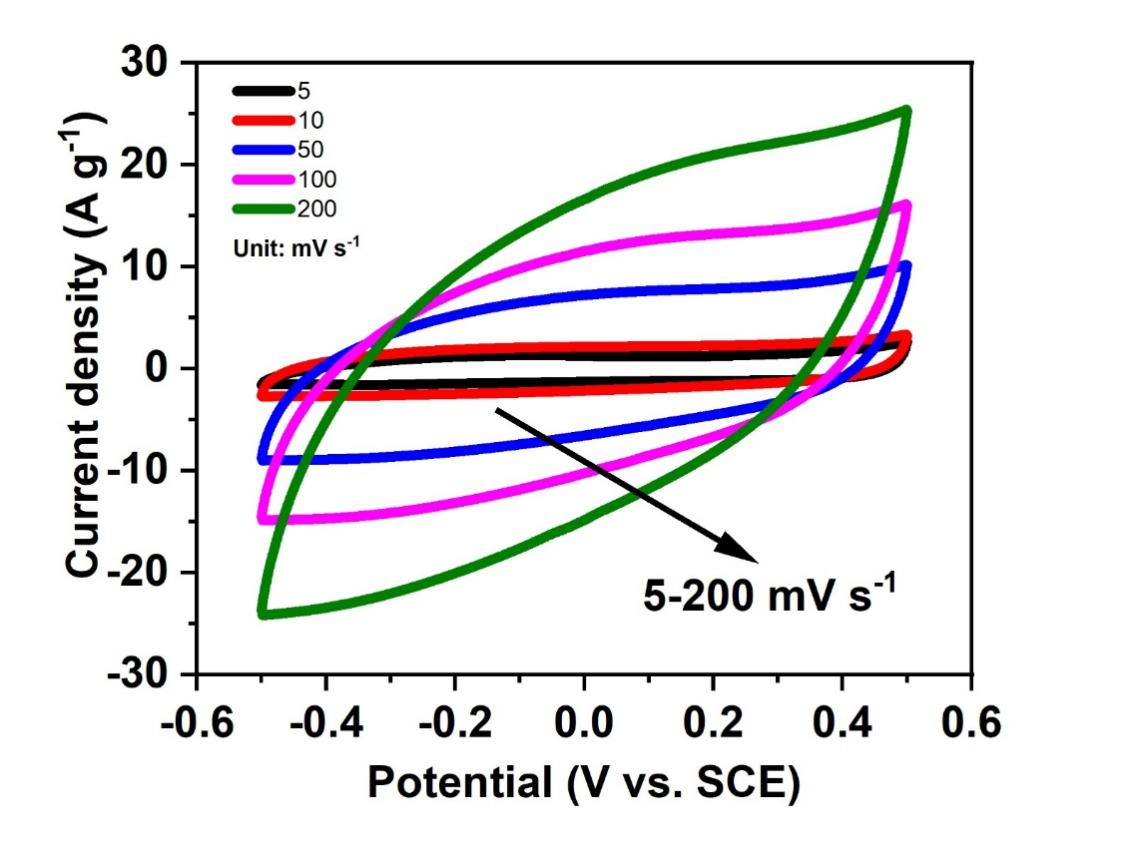


**Figure S11.** CV curves of mPPy/rGO tested at different scan rates from 5–200 mVs^–1^.


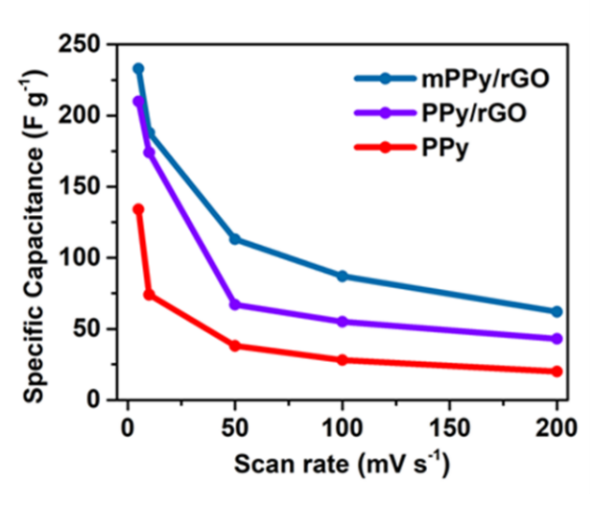


**Figure S12.** Specific capacitance of mPPy/rGO, PPy/rGO and PPy as a function of scan rate ranging from 5 to 200 mVs^–1^.


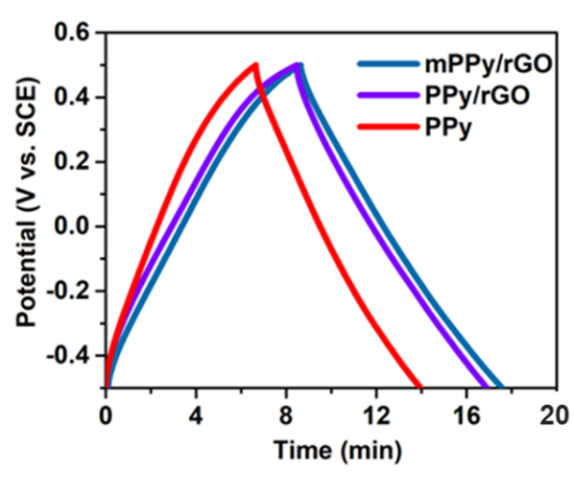


**Figure S13.** GCD curves for mPPy/rGO, PPy/rGO and PPy at the current density of 0.5 A g^–1^.


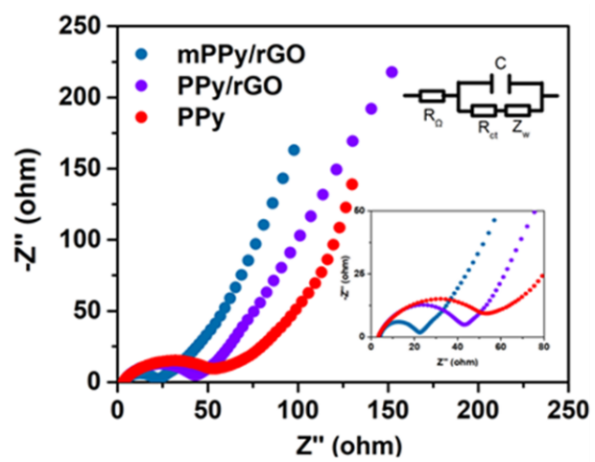


**Figure S14.** Nyquist plots of mPPy/rGO, PPy/rGO and PPy. The inset shows the equivalent circuit model.


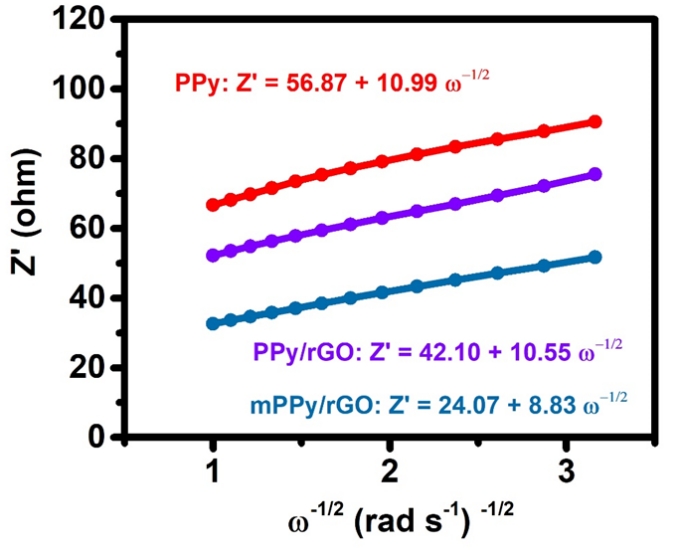


**Figure S15.** The plot of the relationship between Z' and the square root of frequency (w^–1/2^) at low–frequency region for mPPy/rGO (blue), PPy/rGO (purple) and PPy (red).


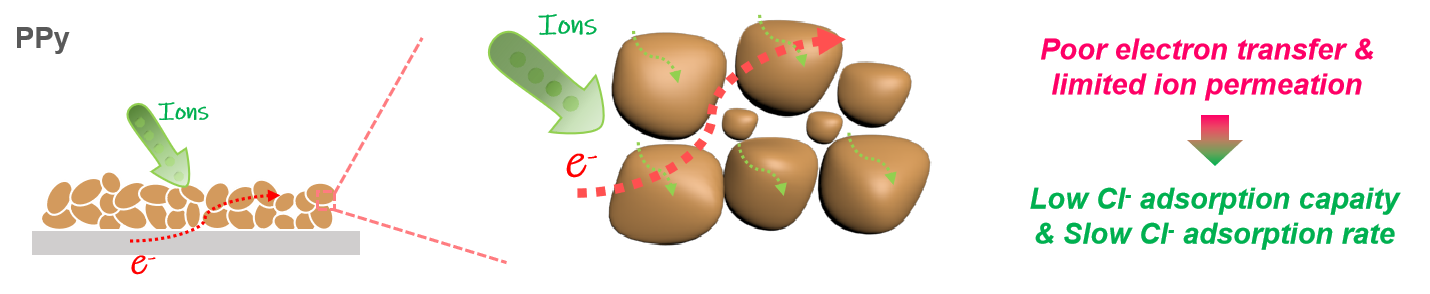


**Figure S16.** Scheme of ion and electron transfer pathways within PPy.

**
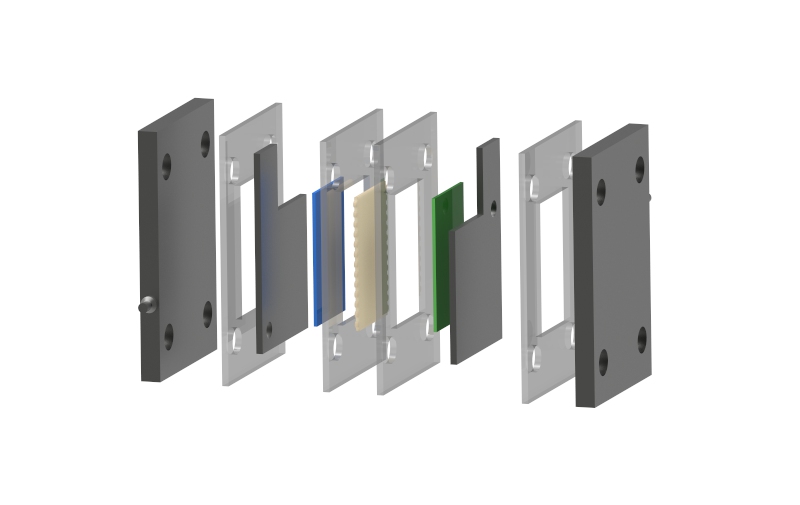
**

**Figure S17.** Scheme of components in a CDI cell, from left to right which are plastic plate, spacer, current collector coated with active materials, cation exchange membrane, spacer, insulation mesh, spacer, anion exchange membrane, current collector coated with active materials, spacer, and plastic plate.


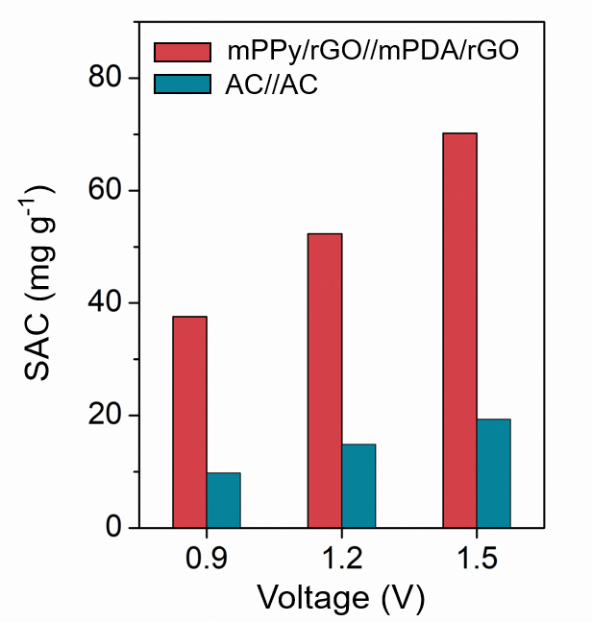


**Figure S18.** SACs of AC-based CDI cell at different voltages from 0.9 – 1.5 V (Concentration: 500 mg L^-1^ NaCl, Flow rate: 20 mL min^-1^).

**S3. Supporting Table S1**

Table S1. The comparison of the desalination capacity of mPPy/rGO with those of some reported materials.

| Materials | SAC (mg g^–1^) | NaCl Concentration (mg L^–1^) | Voltage (V) | Ref. |
| --- | --- | --- | --- | --- |
| mPPy/rGO | 39.3 | 250 | 1.2 | This work |
|  | 52.3 | 500 |  |  |
|  | 70.2 | 1000 |  |  |
|  | 84.1 | 2000 |  |  |
| Graphene sponge | 14.9 | 500 | 1.2 | ^[S1]^ |
| MOF–derived carbon | 15.1 | 292.2 | 1.2 | ^[S2]^ |
| MnO_2_ | 14.9 | 500 | 1.4 | ^[S3]^ |
| MXene | 13 | 292.2 | 1.2 | ^[S4]^ |
| MOF | 11.34 | 584 | 1.2 | ^[S5]^ |
| NiHCF/rGO | 22.8 | 500 | 0.6 | ^[S6]^ |
| MoS_2_ | 25 | 500 | 0.8 | ^[S7]^ |
| DDAQ–based COF | 22.8 | 500 | 1.6 | ^[S8]^ |
| Covalent triazine–based frameworks | 29.34 | 1000 | 1.2 | ^[S9]^ |
| Graphene–polypyrrole–Mn composites | 18.4 | ~500 | 2.0 | ^[S10]^ |
| PPy/activated microporous carbon | 26.6 | 1168.8 | 1.2 | ^[S11]^ |

**References**

[S1] X. Xu, L. Pan, Y. Liu, T. Lu, Z. Sun, D. H. C. Chua, Sci. Rep. 2015, 5, 8458.

[S2] S. Porada, L. Borchardt, M. Oschatz, M. Bryjak, J. S. Atchison, K. J. Keesman, S. Kaskel, P. M. Biesheuvel, V. Presser, Energy & Environ. Sci. 2013, 6, 3700-3712.

[S3] T. Wu, G. Wang, S. Wang, F. Zhan, Y. Fu, H. Qiao, J. Qiu, Environmental Science & Technology Letters 2018, 5, 98-102.

[S4] P. Srimuk, F. Kaasik, B. Krüner, A. Tolosa, S. Fleischmann, N. Jäckel, M. C. Tekeli, M. Aslan, M. E. Suss, V. Presser, J. Mater. Chem. A 2016, 4, 18265-18271.

[S5] Z. Wang, X. Xu, J. Kim, V. Malgras, R. Mo, C. Li, Y. Lin, H. Tan, J. Tang, L. Pan, Y. Bando, T. Yang, Y. Yamauchi, Mater. Horiz. 2019, 6, 1433-1437.

[S6] Z. Ding, X. Xu, Y. Li, K. Wang, T. Lu, L. Pan, Desalination 2019, 468, 114078.

[S7] P. Srimuk, J. Lee, S. Fleischmann, S. Choudhury, N. Jäckel, M. Zeiger, C. Kim, M. Aslan, V. Presser, J. Mater. Chem. A 2017, 5, 15640-15649.

[S8] Y. Li, Z. Ding, X. Zhang, J. Li, X. Liu, T. Lu, Y. Yao, L. Pan, J. Mater. Chem. A 2019, 7, 25305-25313.

[S9] D. Liu, X.-a. Ning, Y. Hong, Y. Li, Q. Bian, J. Zhang, Electrochim. Acta 2019, 296, 327-334.

[S10] X. Gu, Y. Yang, Y. Hu, M. Hu, J. Huang, C. Wang, J. Mater. Chem. A 2015, 3, 5866-5874.

[S11] F. Ji, L. Wang, J. Yang, X. Wu, M. Li, S. Jiang, S. Lin, Z. Chen, J. Mater. Chem. A 2019, 7, 1768-1778.
